# Nonbacterial Thrombotic Endocarditis in a 78-Year-Old Female With Recurrent Acute Ischemic Stroke: A Case Report

**DOI:** 10.7759/cureus.70887

**Published:** 2024-10-05

**Authors:** William R Rankin, Lauren B Querin, Megan Free, Wayne A Martini

**Affiliations:** 1 Emergency Medicine, Mayo Clinic Alix School of Medicine, Scottsdale, USA; 2 Emergency Medicine, Mayo Clinic Arizona, Phoenix, USA; 3 Emergency Medicine, Valleywise Health Medical Center, Phoenix, USA

**Keywords:** acute ischemic infarct, acute myeloid leukemia, anticoagulation, cerebro-vascular accident (stroke), embolic stroke, endocarditis, libman-sacks endocarditis, marantic endocarditis, thromboembolism

## Abstract

Nonbacterial thrombotic endocarditis (NBTE) is a rare but serious complication, particularly in patients with malignancies like acute myeloid leukemia (AML), where a hypercoagulable state increases the risk of embolic events. This case report describes a rare and complex presentation of marantic endocarditis in a 78-year-old female with relapsed AML. The uniqueness of this case lies in the intersection of a hypercoagulable state induced by AML and the resultant NBTE, leading to recurrent embolic strokes, despite oral anticoagulation. This case contributes to the scientific literature by highlighting the diagnostic and therapeutic challenges in managing NBTE in patients with hematologic malignancies. The patient had multiple ED visits, initially with concerns of persistent back spasms, and was diagnosed with venous thromboembolism and started on apixaban.​ She then returned to our ED with visual disturbances and headaches. Significant clinical findings included right hemianopsia and circulating blasts on laboratory tests, indicative of AML relapse. Imaging studies revealed multiple small acute cerebral infarctions and evidence of thrombus affecting the mitral and aortic valves. The patient was diagnosed with NBTE and treated with systemic anticoagulation using warfarin, bridged with enoxaparin sodium. She remained stable during hospitalization and was discharged with follow-up care coordinated among her oncology, neurology, and cardiology teams. NBTE is an important consideration in a differential diagnosis in patients with cancer causing a hypercoagulable state such as AML, particularly in patients who present with neurological symptoms. High index of suspicion and a multidisciplinary approach are essential for the timely diagnosis and management of NBTE to prevent further thromboembolic complications.

## Introduction

Nonbacterial thrombotic endocarditis (NBTE) is a rare, noninfectious condition that causes sterile vegetations on the heart’s valve leaflets. These vegetations, typically composed of fibrin and platelets, are formed when there is a valvular endothelial injury and can result in devastating thrombotic events [1-3]. Nonbacterial thrombotic endocarditis is also known as Libman-Sacks endocarditis or marantic endocarditis. Due to the hypercoagulable state that occurs with malignancy, 39.4%-71.4% of cases of NBTE had an association with underlying cancer, although still a relatively uncommon complication overall [4]. This condition can also be seen in chronic inflammatory conditions including systemic lupus erythematosus (SLE), antiphospholipid syndrome (APS), and myeloproliferative disorders [5,6]. While NBTE is often found incidentally or on post-mortem examination, the most common clinical manifestations are secondary to embolisms.

Endothelial damage from malignancy, driven by cytokine release from macrophages interacting with malignant cells, promotes fibrin and platelet aggregation, leading to NBTE [7,8]. In SLE, turbulence of blood flow combined with chronic deposition of immunoglobulins and complement factors cause immune-mediated endothelial injury [5,7]. Autoantibodies against phospholipids in the cell membrane are the cause of endothelial injury in patients with APS [5,7,9].

## Case presentation

This case report details the clinical course of a 78-year-old female with a complex medical history, including acute myeloid leukemia (AML), status post-coronary artery bypass grafting and stent placement, hyperlipidemia, hypothyroidism, and a history of deep vein thrombosis, and pulmonary embolism. After the initial AML diagnosis, the patient was initially treated with one cycle of decitabine and venetoclax, which resulted in initial remission. However, three months after achieving remission, she experienced a myocardial infarction and, approximately one year later, was found to have a relapse of AML with plans for further chemotherapy.

Shortly after the diagnosis of relapse, the patient presented to the emergency department at a tertiary care academic hospital with a chief complaint of back spasms that had persisted for six days. A chest X-ray performed in the emergency department revealed bibasilar patchy infiltrates, prompting further investigation. Computed tomography (CT) angiogram of the pulmonary arteries revealed small right lower lobe pulmonary emboli and venous ultrasounds detected partially occlusive thrombi in the right peroneal and left posterior tibial and peroneal veins. She was briefly admitted at that time and ultimately discharged four days later on anticoagulation therapy with apixaban.

Approximately one week later, the patient returned with new-onset visual disturbances described as floaters and transient vision loss in the right eye, as well as persistent headaches. These symptoms began after the initiation of apixaban. Physical examination revealed homonymous hemianopsia with loss of the right visual field, although no other focal neurological deficits were noted. The patient’s National Institutes of Health Stroke Scale (NIHSS) score was 2, accounting for the visual field deficit (right homonymous hemianopsia). Laboratory tests revealed circulating blasts consistent with her relapsed AML.

**Figure 1:**
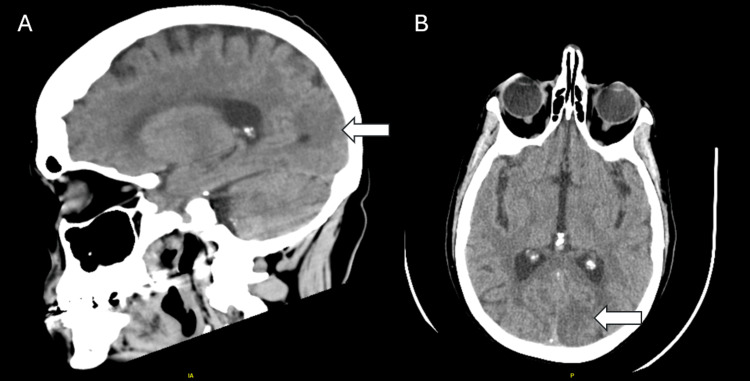
Loss of gray-white matter differentiation in the left parasagittal occipital lobe (arrow) consistent with acute infarct present on both sagittal (A) and axial views (B).

A CT head and neck angiogram was obtained in the ED which revealed an acute left occipital lobe infarct with no significant stenosis, dissection, or aneurysm (Figure 1). An MRI of the brain confirmed multiple small acute infarctions in the perirolandic regions bilaterally and in the left occipital and frontal lobes. Further diagnostic evaluation with a transthoracic echocardiogram (TTE) showed a left ventricular ejection fraction of 57%, with regional wall motion abnormalities and mildly enlarged left atrial size, as well as findings suspicious for vegetation of the aortic valve. A transesophageal echocardiogram (TEE) revealed evidence of marantic endocarditis affecting both the mitral and aortic valves (Video 1), along with a large right atrial wall echodensity suggestive of a thrombus.

**Video 1:** Transesophageal Echocardiogram showing vegetations on both mitral and aortic valves.

With blood culture-negative valvular vegetations and recurrent embolic disease, the patient was given the diagnosis of NBTE and was managed with systemic anticoagulation using warfarin, bridged with enoxaparin. Her condition was monitored with frequent neurological checks, and her anticoagulation therapy was adjusted based on international normalized ratio (INR) results. The patient’s headache was managed with a migraine cocktail consisting of 15 mg of intravenous (IV) ketorolac, 50 mg of IV diphenhydramine, and 5 mg of IV prochlorperazine. During her hospital stay, no new neurological deficits were observed, and follow-up imaging showed stability in infarct size without evidence of hemorrhagic conversion. The patient was ultimately discharged in stable condition, with plans for follow-up with her oncology, neurology, and cardiology teams.

## Discussion

This case emphasizes the importance of considering NBTE in patients with malignancy who present with acute neurological symptoms, particularly when there is already a history of recent thromboembolic events. NBTE is a rare but devastating cause of embolic stroke and other embolic syndromes, including pulmonary embolism, and mesenteric or splenic infarctions. Although 54% of patients with NBTE may present with stroke, its diagnosis can be challenging due to sometimes subtle clinical presentation and the need for specialized imaging techniques, such as TEE, which is the gold standard for the detection of sterile vegetation on cardiac valves. Failure or delay in identification of subtle sterile vegetations and administration of appropriate anticoagulants can lead to devastating embolic events [4].

One of the key strengths in managing this case was the timely decision to use TTE and TEE to confirm the diagnosis of NBTE. Nonbacterial thrombotic endocarditis vegetations are composed of fibrin and platelets, lacking the infectious components seen in infective endocarditis. Transesophageal echocardiogram should be strongly considered when clinical suspicion of NBTE is high, especially if TTE results are inconclusive. Transesophageal echocardiogram can provide detailed information on the size, shape, and mobility of the vegetations, as well as their precise location on the valve leaflets [10,11]. Transesophageal echocardiogram’s enhanced imaging capabilities allow for better visualization of these vegetations, which can be missed by TTE due to its lower spatial resolution [12]. Typical NBTE presents on the mitral and aortic valves along coaptation lines without destruction of valvular tissue, while infective endocarditis is more likely to infect in a unilateral fashion [2]. This detailed imaging is crucial for differentiating NBTE from other forms of endocarditis and for guiding subsequent management decisions.

The management of NBTE in the context of relapsed AML is particularly challenging due to the patient’s hypercoagulable state and the risks associated with anticoagulation therapy. The incidence of thrombotic events in AML patients is notable, with studies reporting venous thromboembolism rates of approximately 12.3% and arterial thromboembolism rates of 2.3% [13]. Additionally, NBTE vegetations are more friable and predisposed to embolization when compared with infective endocarditis [4].

Anticoagulation in cancer patients, including those with AML, is essential for managing thrombotic risks but carries significant bleeding risks. Approximately 40.5% of NBTE cases are associated with an underlying malignancy [12]. Cancer patients, particularly those with hematologic malignancies like AML, have an increased risk of bleeding compared to patients without cancer due to factors such as thrombocytopenia and the effects of intensive myelosuppressive chemotherapy. Balancing the risks of thrombosis and bleeding is particularly challenging in this population [14,15].

In AML patients with NBTE, anticoagulation is necessary to prevent thromboembolic events, but the heightened bleeding risk must be carefully managed. Low molecular weight heparin is often preferred due to its efficacy and safety profile, but the choice of anticoagulant must be individualized based on the patient's bleeding risk and other clinical factors [15].

In this case, the patient’s AML relapse created a hypercoagulable state, which contributed to the development of NBTE and ultimately recurrent embolic strokes. This patient was successfully managed with warfarin, with careful monitoring of INR and adjustment of therapy to prevent further embolic events. Detection of such cases can be challenging, as the presentation of visual disturbances and headaches could have been attributed to a wide variety of causes. Clinical clues include negative blood cultures, a normal thrombophilic screen, and absence of clinical infection signs [4]. This case underscores the need for a multidisciplinary approach in the management of such complex cases, involving oncology, cardiology, and neurology teams to optimize patient outcomes.

## Conclusions

Nonbacterial thrombotic endocarditis is a critical but often underrecognized cause of recurrent embolic events in patients with malignancies or other systemic inflammatory conditions. Emergency physicians should maintain a high level of suspicion for this considering patients with a history of cancer who present with multiple thrombotic events including acute cerebrovascular accidents. Early recognition, often requiring transesophageal echocardiogram, and appropriate management, including initiation of systemic anticoagulation therapy, are essential to prevent further complications and improve patient outcomes.
